# Hematological, clinical, cytogenetic and molecular profiles of confirmed chronic myeloid leukemia patients at presentation at a tertiary care teaching hospital in Addis Ababa, Ethiopia: a cross-sectional study

**DOI:** 10.1186/s12885-024-12282-x

**Published:** 2024-04-26

**Authors:** Fekadu Urgessa, Boki Lengiso, Aster Tsegaye, Amha Gebremedhin, Fozia Abdella, Fisihatsion Tadesse, Jerald Radich, Helen Nigussie, Teklu Kuru Gerbaba

**Affiliations:** 1 Medical Laboratory Sciences Department, College of Health Sciences, Addis Ababa University, Addis Ababa, Ethiopia; 2 Internal Medicine Department, College of Health Sciences, Addis Ababa University, Addis Ababa, Ethiopia; 3 Cellular and Molecular Biology Department, College of Natural and Computational Sciences, Addis Ababa University, Addis, Ababa Ethiopia; 4 Fred Hutchison Cancer Center, Seattle, WA USA; 5 Microbix Biosystems Inc, Mississauga, ON, Canada; 6 Ethiopian Public Health Institute, Addis Ababa, Ethiopia

## Abstract

**Background:**

In low-income countries there is insufficient evidence on hematological, clinical, cytogenetic and molecular profiles among new CML patients. Therefore, we performed this study among newly confirmed CML patients at Tikur Anbesa Specialized Hospital (TASH), Ethiopia.

**Objective:**

To determine the hematological, clinical, cytogenetic and molecular profiles of confirmed CML patients at tertiary care teaching hospital in Addis Ababa, Ethiopia.

**Methods:**

A facility-based cross-sectional study was conducted to evaluate hematological, clinical, cytogenetic and molecular profiles of confirmed CML patients at TASH from August 2021 to December 2022. A structured questionnaire was used to collect the patients’ sociodemographic information, medical history and physical examination, and blood samples were also collected for hematological, cytogenetic and molecular tests. Descriptive statistics were used to analyze the sociodemographic, hematological, clinical, cytogenetic and molecular profiles of the study participants.

**Results:**

A total of 251 confirmed new CML patients were recruited for the study. The majority of patients were male (151 [60.2%]; chronic (CP) CML, 213 [84.7%]; and had a median age of 36 years. The median (IQR) WBC, RBC, HGB and PLT counts were 217.7 (155.62–307.4) x10^3^/µL, 3.2 (2.72–3.6) x10^6^/µL, 9.3 (8.2–11) g/dl and 324 (211–499) x 10^3^/µL, respectively. All patients had leukocytosis, and 92.8%, 95.6% and 99.2% of the patients developed anemia, hyperleukocytosis and neutrophilia, respectively. Fatigue, abdominal pain, splenomegaly and weight loss were the common signs and symptoms observed among CML patients. Approximately 86.1% of the study participants were Philadelphia chromosome positive (Ph+) according to fluorescence in situ hybridization (FISH). P210, the major breakpoint protein, transcript was detected by both qualitative polymerase chain reaction (PCR) and quantitative real time polymerase chain reaction (PCR).

**Conclusion:**

During presentation, most CML patients presented with hyperleukocytosis, neutrophilia and anemia at TASH, Addis Ababa. Fatigue, abdominal pain, splenomegaly and weight loss were the most common signs and symptoms observed in the CML patients. Most CML patients were diagnosed by FISH, and p120 was detected in all CML patients diagnosed by PCR. The majority of CML patients arrive at referral center with advanced signs and symptoms, so better to decentralize the service to peripheral health facilities.

## Background

Chronic myeloid leukemia (CML) is a hematopoietic stem cell (HSC)-derived myeloproliferative disorder caused by chromosomal translocation (9; 22). The formation of the Philadelphia (Ph) chromosome and the expression of the breakpoint cluster region (BCR)-Abelson murine leukemia viral oncogene homolog 1 (ABL) fusion gene result from the fusion of a portion of the BCR on chromosome 22 with the ABL tyrosine kinase on chromosome 9 [1, 2]. Cytogenetic evidence of the Philadelphia (Ph) chromosome, a reciprocal translocation between the long arm of chromosome 22 at the BCR gene and chromosome 9 at the ABL gene, can be found in more than 95% of CML patients (9;22). As a result, a BCR-ABL fusion gene is formed, resulting in the production of a chimeric protein with constitutively active tyrosine kinase activity [2–5].

CML is characterized by anemia, thrombocytosis, and left-shifted leukocytosis [6]. Most cases of CML can be diagnosed using peripheral blood findings combined with molecular genetic techniques that detect t(9; 22) (q34.1; q11.2) or, more specifically, BCR::ABL1. A bone marrow aspirate, on the other hand, is required to ensure sufficient material for a complete karyotype and for morphologic evaluation to confirm the disease phase [7]. Patients in the chronic phase of CML have less than 10% blasts in their bone marrow samples [8]. The accelerated phase of CML is distinguished by bone marrow samples containing 10–20% blasts. The CML blast crisis phase has the same picture as acute leukemia; this stage contains more than 20% blasts, and large clusters of blasts are observed in the bone marrow and spread to tissues and organs other than the bone marrow [8, 9].

Molecular tests such as PCR and FISH for t(9;22) (q34;q11.2), which demonstrate BCR-ABLs, are essential for the diagnosis and confirmation of CML [10, 11]. The goals of treatment for chronic phase CML are to avoid progression to other advanced stages and to avoid adverse events (AEs) to restore and maintain quality of life so that patients can achieve a life expectancy comparable with that of the general population [12]. Hydroxyurea (HU) is an S-phase agent that acts by inhibiting DNA synthesis. This drug acts as an inhibitor of ribonucleotide reductase and can lower blood counts within 1 to 2 days, especially if higher than standard doses are used [13]. Tyrosine kinase inhibitors (TKIs) are potent drugs that significantly improve long-term outcomes in patients with CML [14].

In the developed world, early diagnosis at an average age of approximately 65 years is common, and most cases are chronic. In contrast, in low-income countries, CML is more commonly diagnosed in the advanced stage and in the younger age group (39 years) [3]. Early diagnosis and treatment of CML are highly important for preventing and controlling CML as its diagnosis and treatment delay worsen because the disease may progress to clonal mutation and the development of resistance to TKI therapy [13]. Besides, in low-income countries there is insufficient evidence on hematological, clinical, cytogenetic and molecular profiles among new CML patients. Ethiopia is among the low-income and resource-constrained countries where there is limited evidence on the status of new CML patients. Thus, the aim of this study was to determine the hematological, clinical, cytogenetic and molecular profiles of confirmed CML patients during their first presentation at TASH, Addis Ababa, Ethiopia.

## Methodology

The study was conducted at TASH hematology clinics, a tertiary care center affiliated with the College of Health Sciences, Addis Ababa University (AAU). TASH was the largest referral hospital in Ethiopia. Patients with CML from all around the country were sent to this institution to be enrolled in the Glivec International Patient Assistance Program and receive treatment (GIPAP).

A cross-sectional study was conducted among confirmed chronic myeloid leukemia patients who were first diagnosed at TASH between August 2021 and December 2022. The study population included CML patients who were diagnosed and confirmed by Xpert BCR-ABL Ultra, FISH and RT‒PCR as CML patients for the first time at TASH hematology clinics. Excluded patients included those with suspected CML who were BCR-ABL, FISH and RT‒PCR negative and confirmed to be CML negative; patients who did not voluntarily provide blood samples; and patients who were extremely ill and unable to provide consent and samples.

A convenient sampling technique was used for all the newly diagnosed patients enrolled during the study period. All CML patients diagnosed between August 2021 and December 2022 were included consecutively to determine the hematological, clinical, cytogenetic and molecular profiles. Accordingly, 251 CML patients were recruited for the study.

### Sample collection and laboratory analysis

Blood samples with a volume of 5 mL were collected in an EDTA test tube from all study participants by a certified laboratory expert. Hematological analysis was performed using a Unicel DxH800 at TASH according to the SOPs within a maximum of 8 h following collection. The CBC was analyzed with a Beckman Coulter DxH 800 automated hematology analyzer based on the manufacturer’s recommendations.

For molecular profiles, one of the results used for confirming CML was the Xpert BCR-ABL Ultra test. In vitro diagnostic tests for the quantitation of BCR::ABL1 and ABL1 mRNA transcripts in peripheral blood specimens from patients diagnosed with t(9;22)-positive CML expressing the BCR-ABLl fusion transcripts type e13a2 and/or e14a2 have been performed. The test utilizes automated, quantitative, RT‒qPCR, but it could not differentiate e13a2 from e14a2. The Xpert BCR-ABL Ultra test is intended to measure BCR::ABL1-to-ABL1% ratios on the International Scale (IS).

The other test used for the study was the FISH technique for the initial screening of CML patients via both bone marrow and peripheral blood. The RT‒PCR results were also consistent with the results of other tests we used for the present study, and these results constitute the gold standard for diagnosing and monitoring BCR::ABL1 transcript mRNA in CML patients. All FISH, qualitative and quantitative RT‒PCR were performed abroad in India for all patients.

### Quality assurance

The overall activities of data collection were monitored by the principal investigator to maintain the validity of the data during data collection. There was strict supervision during the data collection, and the collected data were checked for completeness, accuracy, clarity, and consistency by the supervisors on a daily basis and by the principal investigator. The samples were verified for hemolysis, clotting, volume, collection time, and proper labeling before proceeding to the analytical procedure. The manufacturer’s procedures, as well as safety precautions and specimen handling procedures, were strictly followed.

The analysis was performed by a senior laboratory technologist. Standard operating procedures were used for specimen processing to maintain good quality. The performance of the automated hematology analyzer was checked by running three levels of hematological cell controls (normal, low and high). The results of complete blood counts were registered as the exact number (value) in a standardized recording format. All laboratory assays were carried out following standard operating procedures (SOPs) by trained and experienced medical technologists.

The data collected were cleaned, checked for completeness and entered in Excel spreadsheet. The data were subsequently exported to the Statistical Package for Social Sciences (SPSS) version 26. The data were analyzed using SPSS. Descriptive statistics were used to analyze the sociodemographic, hematological, clinical, cytogenetic and molecular profiles of the study participants. The distribution of the data was checked, and the median and IQR were used for non-normally distributed data.

### Operational definitions

#### Anemia

Hgb value < 13 g/dl for males and < 12 g/dl for females. Severe anemia ≤ 7 g/dl, moderate anemia 7.01–10 g/dl, mild anemia 10.01–12 g/dl for females and 10.01–13 g/dl for males.

#### Basophilia

when a basophil count > 300 cells/µL is found in blood.

#### Clinical profile

Signs and symptoms of CML patients during presentation.

#### Confirmed new CML patient

Xpert BCR-ABL Ultra or FISH- or RT‒PCR-positive patients confirmed for the first time as CML patients at TASH hematology clinics.

#### Eosinophilia

when the eosinophil count is > 500 cells/µL in blood.

Hyperleukocytosis was defined as a WBC greater than 100,000/µL found in blood.

**Neutrophilia** was defined as a higher neutrophil count (> 7.9 × 103/µL). Prechemotherapy naive if the patients never received HU treatment

**Pre**chemotherapy treated Patients who had received HU treatment

#### Splenomegaly

Spleen swelling of the categorized size; massive splenomegaly > 10 cm, moderate 4–9 cm and mild size of 1–3 cm.

#### Thrombocytopenia

a PLT < 150 × 10^3^ cells/µL found in blood.

#### Thrombocytosis

a PLT > 450 × 10^3^ cells/µL found in blood.

## Results

### Sociodemographic characteristics of the study participants

A total of 251 CML patients were diagnosed and included in the study. Among the total study participants, 107 and 144 were naïve and HU-treated, respectively. More than half (60.2%, 151) of the patients were males. The median age of the study participants was 36 (IQR (29–47) years, and more than 91% of the study participants were ≤ 60 years old. More than 51% and 44% came from rural area and travelled more than 300KM distance to reach the TASH, respectively. The median for duration with symptoms of our study participant was five months (Table 1). And the time it takes from initial diagnosis to confirmation was approximately two weeks since most of the patients should send the sample to abroad for confirmation.

**Table 1:** Socio-demographic characteristics of confirmed CML patients during presentation (n = 251)

Variables	Category	N (%)
Age	In year, Median (IQR)	36 (29–47)
Sex	Male	151(60.2)
	Female	100(39.8)
Residence	Urban	122(48.6)
	Rural	129(51.4)
Educational background	Can’t read and write	68(27.1)
	Primary Education	95(37.9)
	Secondary Education	44(17.5)
	Higher Education	44(17.5)
Occupation	Farmer	93(36.3)
	House Wife	28(10.9)
	Gov’t employee	18(7.0)
	Student	20(7.8)
	Merchant	20(7.8)
	Private employee	18(4.7)
	Driver	10(3.9)
	Construction Related	9(3.5)
	Military	4(1.6)
	Others	31(12.4)
Pre-chemo treatment (hydroxyurea)	Yes	144 (57.4)
	No, Naïve	107(42.6)
Approximate distance between patients’ home and TASH	Less than 100 km	67(26.7)
	100–300 km	73(29.1)
	More than 300 km	111 (44.3)
Marital Status	Single	57(22.7)
	Married	185(73.7)
	Divorced	6(2.4)
	Widowed	3(1.2)
Duration of symptoms	In, month, Mean (IQR)	5(2.25-12)
	In month, Range	0–60

### Hematological profile of the study participants

The median (IQR) WBC count was 217.7 (155.62–307.4) ×10^3^/µL, which was determined for patients who were both naïve and treated with HU during presentation. The median (IQR) WBC counts for patients who were naïve and treated with HU were 261.2 (186.6-349.7) × 10^3^/µL and 188.3 (139.3-290.73) ×10^3^/µl, respectively, during presentation. For the absolute neutrophil count (ANC), the overall median (IQR) was 174.8 (111.3-263.6) x10^3^/µL, whereas for patients naive and treated with HU, the median (IQR) was 201 (137.12–295.6) x10^3^/µL and 145.25 (97.2-241.57) x10^3^/µL, respectively.

For RBC parameters, the median (IQR) value for RBC was 3.2 (2.72–3.6) x10^6^/µL. The median (IQR) RBC counts for patients who were naïve to treatment and those treated with HU were 3.05 (2.7–3.6) ×10^6^/µL and 3.3 (2.72–3.75) ×10^6^/µL, respectively. With respect to the PLT, the median (IQR) value was 324 (211–499) x10^3^/µL. The median (interquartilerange (IQR)) PLTs for patients naïve and treated with HU were 322 (211–447) ×10^3^/µL and 324.5 (211-508.75) ×10^3^/µL, respectively. There were significant differences in hematological parameters, such as the WBC count (*P < 0.001*), HGB count (*P = 0.024*), and HCT count (*P = 0.024*), between naive and HU treated groups (Table 2).

**Table 2:** Hematological profile of confirmed CML patients at presentation (*n* = 251)

Hematological parameters	Total (Median, IQR)	Prechemo treatment (Hydroxyurea)		P value
		Naïve (Median, IQR)	Treated (Median, IQR)	
WBC (x103/µL)	217.7(155.62–307.4)	261.2(186.6-349.7)	188.3(139.3-290.73)	<0.001
RBC (x106/µL)	3.2(2.72–3.6)	3.05(2.7–3.6)	3.3(2.72–3.75)	0.153
HGB (g/dl)	9.3(8.2–11)	8.9(7.9–10.2)	9.75(8.33–11.2)	0.024
HCT (%)	29.05(25.85–33.5)	28.3(24.3–32.2)	29.75(26.75–34.4)	0.024
MCV (fL)	92.85(87.3–97.2)	92.5(86.9–96.3)	93.05(88-98.56)	0.217
MCH (pg)	29.5(27.8–31.4)	29.1(27.5–30.7)	29.9(27.97–31.85)	0.061
MCHC (g/dl)	31.6(30.8–32.6)	31.6(30.6–32.6)	31.6(30.9–32.6)	0.538
RDW (%)	20.8(19.5–23.3)	20.6(19.4–22.2)	21.2(19.5–23.4)	0.304
PLT (x103/µL)	324 (211–499)	322(211–447)	324.5(211-508.75)	0.592
MPV (fL)	8.9 (8.1–9.8)	8.8 (8.07–9.3)	9 (8.2–10)	0.628
NE %	85.75 (78.1-89.77)	87.4 (80.9–90.3)	84.3 (77.8–88.4)	0.001
LY %	4.5 (2.53–7.3)	3.8 (2.2–6.83)	5.2 (2.9–7.6)	0.008
MO %	4.25 (2.8–6.5)	3.5 (2.5–5.7)	4.7 (3.07–6.8)	0.005
EO %	2.3 (1.4–3.85)	2.4 (1.5–3.6)	2.3 (1.2–4.05)	0.557
BA %	1.1 (0.3–3.3)	0.8 (0.3-2)	1.4 (0.45–4.7)	0.002
NE # x103/µL	174.8(111.3-263.6)	201(137.12–295.6)	145.25(97.2-241.57)	0.000
LY # x103/µL	7.85(4.95–14.3)	7.52(5.1-14.15)	8.2(4.7–15.3)	0.975
MO # x103/µL	9.2(4.9-14.12)	9.1(4.85–13.15)	9.4(4.97–15.35)	0.926
EO # x103/µL	5.1(2.7–8.8)	5.95(3.0-10.23)	4.1(2.1–8.2)	0.004
BA # x103/µL	2.39(0.53–6.3)	2.1(0.5–5.7)	2.92(0.72–7.5)	0.083

### Common hematological abnormalities among new CML patients

All patients had leukocytosis, and greater than 95.6% of the patients had hyperleukocytosis. Among the study participants, 233 (92.8%) CML patients developed anemia, and moderate anemia was common among 136 (54.2%) CML patients, followed by mild anemia 76 (30.3%) and severe anemia 21 (8.4%) during presentation. The percentages of CML patients with eosinophilia and basophilia were 96.5% and 88.5%, respectively. However, the medians (IQRs) for eosinophils and basophils were 2.3 (1.4–3.85)% and 1.1 (0.3–3.3)%, respectively, of the total WBC count (Table 3).

**Table 3:** Common hematological abnormalities among new CML patients (*n* = 251)

S. No	Hematological Abnormalities	n (%)
1	Leukocytosis	251 (100)
2	Neutrophilia	249 (99.2)
3	Hyperleukocytosis	240(95.6)
4	Anemia	233 (92.8)
5	Eosinophilia	242(96.5)
6	Basophilia	222(88.5)
7	Thrombocytosis	73(29.1)
8	Thrombocytopenia	34 (13.5)

### Distribution of common hematological abnormalities among patients in the CML phases

Among the 91 HUnaïve and 122 HUtreated CP CML patients, 90 (98.9%) and 113 (92.6%) had hyperleukocytosis, respectively. Most CP-phase WBCs fell in the range of 100–250 × 10^3^/µL, whereas 33 (15.5%) CP and 10 (33.3%) AP patients had ≥ 351 × 10^3^/µL WBC counts. In the AP CML phase, 29 (96.7%) patients had hyperleukocytosis, whereas all eight BC CML patients had hyperleukocytosis. Among the 91 HUnaïve and 121 HUtreated CP CML patients, 58 (63.7%) and 55 (45.1%) had moderate anemia, respectively. Among the phases, 89.2% and 90% of the CPs and APs, respectively, developed anemia, whereas only one BC CML patient treated with HU was free from anemia. Regarding the PLT, of the 91 HUnaïve and 121 HUtreated CP CML patients, 54 (59.3%) and 70 (57.4%) had normal PLTs, respectively. Furthermore, thrombocytopenia was observed in 27 (12.7%), 3 (10%) and 4 (50%) patients in the CP, AP and BC CML phases, respectively. Thrombocytosis was observed in 62 (29.1%) patients with CP, 10 (32.2%) with AP and one patient in the BC CML phase. For the neutrophilic distribution, except for one patient in the CP CML phase, there was neutrophilia in all phases of CML.

### Clinical profile of the study participants

**Fig. 1:**
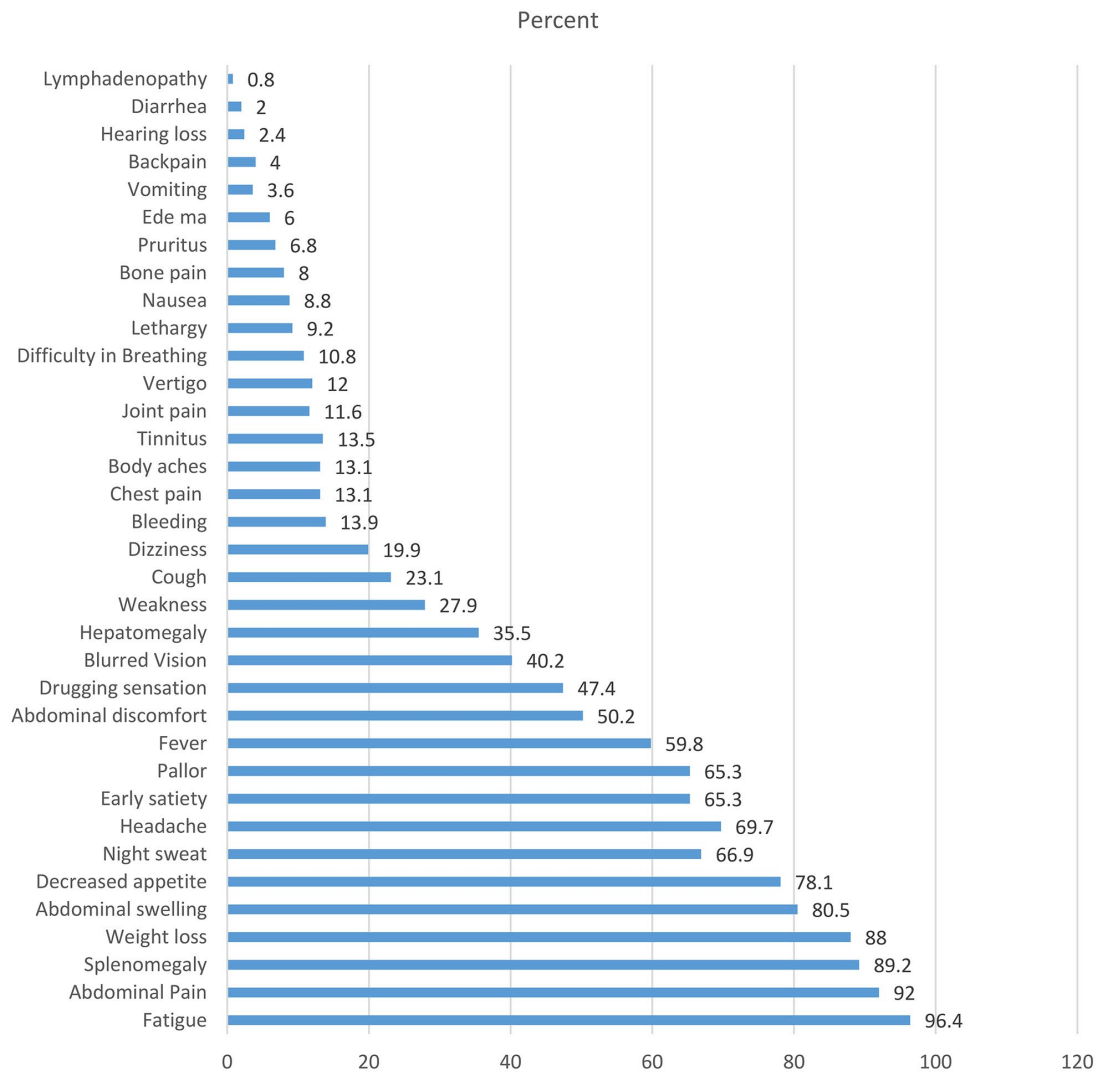
Clinical profile of confirmed CML patients at presentation

Among 251 study participants, 213 (84.7%) were at the CP stage, 30 (12%) were at the AP stage, and 8 (3.2%) were at the BC stage. The majority of CML patients were symptomatic at the time of presentation; the most common symptoms were fatigue (242; 96.4%), abdominal pain (231; 92%), splenomegaly (232; 92.4%) and weight loss (221; 88%) (Fig. 1). The prognostic risk scores of all the study participant were calculated (Table 4).

**Table 4:** Prognostic risk scores among new CML patients (*n* = 251)

Risk Scores	Frequency	Percentage
EUTOS Score		
High Risk	**116**	**46.2**
Low Risk	**135**	**53.8**
Sokal Score		
High Risk	**149**	**59.4**
Intermediate Risk	**98**	**39**
Low Risk	**4**	**1.6**
ELTS Score		
High Risk	**138**	**55**
Intermediate Risk	**97**	**38.6**
Low Risk	**16**	**6.4**

Splenomegaly is one of the main symptoms of patients. A massive, moderate or mild increase in spleen size was indicated. Among the 232 study participants who had splenomegaly, 62.9% had massive splenomegaly with a spleen size > 10 cm, 34.9% had moderate splenomegaly with a spleen size of 4–9 cm, and 2.1% had mild splenomegaly with a spleen size of 1–3 cm. Approximately  85% of CPs had splenomegaly, whereas only one AP and one BP CML patient were free from splenomegaly.

### Cytogenetic and molecular profiles

All of the study participants were positive for the Philadelphia chromosome or BCR::ABL1, and the p210 BCR::ABL1 fusion transcript was detected. After cytogenetic analysis of 251 confirmed CML patients, FISH was performed for 222 (88.5%) CML patients; 216 (97.3%) were Philadelphia chromosome positive (Ph+), whereas in six patients Philadelphia was absent or negative. The median (IQR) number of Ph + cells per 100 cells was 95% (85–100%) among the study participants diagnosed and positive by FISH. Among the Ph + cases, the distributions of CML phases were 85.6%, 11.6% and 2.8% for CP, AP and BC, respectively. Except for one AP patient, all the other Ph-negative patients were CP CML patients.

The remaining 35 (13.9%) patients were diagnosed by the Xpert BCR-ABL Ultra test (GeneXpert, Cepheid) (twety three) (23) and by quantitative (six) and qualitative (six PCR. In our study area, for new patients, the Xpert BCR-ABL Ultra test (RT‒PCR) was performed for patients who cannot afford FISH (which is performed abroad). The Xpert BCR-ABL Ultra test results showed that 20 (87%) of the new CML patients had > 55% (above the upper LoQ), which indicated that BCR::ABL1 L fusion was detected at a level > 55% (IS). Qualitative PCR was performed for six patients who were FISH negative, and p210 (BCR/ABL), the major breakpoint protein, transcript was detected. Among the transcripts identified by both quantitative and qualitative PCR, all the detected transcripts were of the major breakpoint protein, p210 (BCR/ABL), which is frequently encoded by the e13a2/e14a2 isoforms.

## Discussion

CML is characterized by anemia, thrombocytosis, and leukocytosis with a shift to the left. Molecular tests such as PCR and FISH for t(9;22) (q34;q11.2), which demonstrate BCR-ABLs, are essential for the diagnosis and confirmation of CML [10, 11]. However, the data related to hematological, clinical, cytogenetic and molecular profiles of CML patients is very limited in low-income countries as a result we motivated to describe these profiles among CML patients during presentation.

The median age was 36 years, and more than 91% of the study participants were ≤ 60 years old. This finding is consistent with the study conducted by Mulu Fentie A et al., which reported a total of 27.2% of patients aged 31–40 years [15]. These findings are comparable with those of the studies conducted by Tadesse F et al., in which the median age was 33 years [3], and by Ngono AP et al., in which the mean age was 39.2 years [1]. This shows that younger populations were affected by CML. In this study, males (60.2%) were more affected by CML than females were, with a ratio of 1.5:1. This finding is also similar to that of a study conducted in Pakistan, in which the male-to-female ratio was 1.6:1 [16, 17]. This finding is also similar to that of a study conducted in Iraq in which 57.1% of the participants were male (Al-abady I et al., 2021) [18]. These findings may support the finding that CML affects younger individuals aged 39 years in low-income countries [3], although the reason why younger individuals are affected by CML is not clear in developing countries.

Among 251 study participants, 213 (84.8%) were in the CP, 30 (12%) were in the AP and 8 (3.2%) were in the BC. These findings are similar to those of Kumar S et al., who reported that 83% of CML‑CP, 12% of AP, and 5% of AP patients were in the BC phase in India [19]. Furthermore, a study from India by Srinivas KG et al. reported comparable findings: CP (90.1%), AP (4.5%) and BC (5.4%) [20]. However, our findings were inconsistent with those of Ngono AP et al. in Cameroon, especially for CP-CML (66%) [1], and with those of Sinha R. et al. [21] in India. Sinha et al. reported that 28.1% of patients were in the AP phase, which is higher than our finding (12.1%). Similarly, there were fewer findings in the BC phase than in Cameron, 22.7% of which were reported by Ngono AP et al. and 7.8% by Sinha R et al. in India [1, 21]. This difference could be due to the difference in sample size since the sample sizes for both patients were much smaller than our sample size and because of the difference in duration of symptoms. Furthermore, advanced-stage CML was prevalent, possibly because patients were diagnosed at advanced stages because of late detection, the lack of facilities available to detect CML at peripheral health facilities and poor culture available to visit health facilities for health checks.

The median (IQR) WBC count was 217.7 (155.62–307.4) ×10^3^/µL, and all patients had leucocytosis, whereas 95.6% had hyperleukocytosis. This finding is similar to that of the study conducted by Tadesse F et al., which revealed that all the patients had leukocytosis [3]. Similarly, in a study conducted in Iraq by Al-abady I et al., the mean WBC count was 153.7 × 10^9^/L, ranging from 29 to 436 × 109/L. Furthermore, in a study performed in Tanzania by Henke O et al., the median WBC was 300.5 × 10^9^/L (78–499 × 10^9^/L) [22]. This finding was also consistent with those of studies conducted by Ngono AP et al., Kumar et al. and Wiyono et al. [1, 6, 19]. Leukocytosis is expected in CML patients, but as most of the study participants had hyperleukocytosis, this could make diagnosis difficult because the patients need to come to TASH frequently and be forced to take HU for a longer time as the WBC count decreases to the recommended number (20–30 × 10^9^/L and clinical parameters also considered) to start chemotherapy. This could also explain why CML patients dropped out before starting chemotherapy (imatinib, a first generation TKI) in our study area.

For the absolute neutrophil count (ANC), the median (IQR) value was 174.8 (111.3-263.6) x10^3^/µL during presentation. Patients who were naïve to treatment or treated with HU had 201 (137.12–295.6) x10^3^/µL and 145.25 (97.2-241.57) x10^3^/µL ANC, respectively. Our study showed that almost all (99.2%) of the participants had neutrophilia, which is in line with the results reported from Tanzania by Tebuka E et al. (ANC 179(± 123) K/µL) [22]. In addition, our study revealed that 88.5% of the study participants had basophilia, although the relative percentage median (IQR) was 1.1 (0.3–3.3)%, which is comparable with the findings of Al-abady et al. [18]. . Neutrophils are among the WBC components that are expected to increase in CML patients; as a result, neutrophilia was observed in almost all the study participants. In addition, basophil formation is usually observed in CML patients; as a result, basophilia was also prevalent, although the relative percentage is dominated by neutrophils. However, neutrophilia and basophilia were also observed among patients treated with HU because either the patients took HU for short days or they took a low dose during presentation.

For Hgb, the median (IQR) value was 9.3 (8.2–11) g/dl. The median (IQR) values for patients who were naïve to treatment and treated with HU were 8.9 (7.9–10.2) g/dl and 9.75 (8.33–11.2) g/dl, respectively. Approximately 19(7.4%) had severe, 138(53.9%) moderate and 78(30.4%) mild anemia which was comparable with study conducted by Tadesse F et al. that reported 96.7% of CML patients had anemia [3]. These findings are also similar to those of a study by Kumar S *et al., in which* 13% had severe anemia, 58.8% had moderate anemia and 28.2% had mild anemia [19], and those of Ngono AP et al., in which 86.4%, 61.4% and 5.3% had moderate, respectively [1]. Furthermore, a study conducted by Chang F et al. revealed that 95.1% of patients developed anemia and mild anemia (22.8%) and moderate anemia (46.9%), but these proportions differed for severe anemia (30.1%) [16]. The difference may be due to sample size, duration of symptoms and geographical location. As expected, the incidence of anemia was high, and moderate anemia was the dominant anemia among the study participants; this was also related to the clinical stage at which the patients came to our study site, TASH.

With respect to the PLT, the median (IQR) value was 324 (211–499) x10^3^/µL during presentation. The medians (IQRs) of the PLTs for patients’ naïve to treatment with HU were 322 (211–447) ×10^3^/µL and 324.5 (211-508.75) ×10^3^/µL. Approximately 73 (29.1%) and 34 (13.5%) patients had thrombocytosis and thrombocytopenia, respectively. These findings were consistent with those of the study conducted by Ngono AP et al., which reported that a normal PLT was found in 80 (60.6%) patients, 11 (8.3%) had thrombocytopenia, and 41 (31.1%) had thrombocytosis [1]. In addition, our findings were comparable with those of a study conducted by Tadesse et al., which revealed that 23.3% of the study participants had thrombocytosis [3], however, there is some variation, which could be due to sample size differences or variations since our sample size was larger than those in those studies. In addition, this was an indication of the variation in the PLT among CML patients, which could be normal in some cases but thrombocytosis in others.

The duration of symptoms in our study participants (median (IQR)) was 5 (2.25-12) months, with a range from 0 to 60 months. These results were comparable with the results reported from Tanzania by Tebuka E et al., in which the duration of symptoms was 15 (± 9) months, ranging from 0 to 48 months [23]. All CML patients in this study were symptomatic at the time of presentation, and the common complaint was fatigue (96.4%), followed by abdominal pain (92%), splenomegaly (89.2%), weight loss (88%) and abdominal swelling (80.5%). These findings are in line with the findings of studies conducted by Sultan S et al., Al-abady I et al. and Sinha R [17, 18, 21]. This percentage was greater than that in the study by *Srinivas KG et al.*., which revealed that the common presentations were fatigue (60%), fever (48%), weight loss (37%) and splenomegaly (20%). Another Indian study revealed that fullness in the abdomen was 66.6%, fever was 59%, and fatigue was 55.5% [19]; these findings can be attributed to differences in sample sizes among studies, environmental factors and durations of symptoms.

Splenomegaly (92.4%) presented in different categorical sizes in this study; 146 (62.9%) patients had massive splenomegaly, 81 (34.9%) developed moderate splenomegaly, and 5 (2.1%) had mild splenomegaly. These findings are comparable with the study performed by Tadese F et al., whose overall range was 3–26 cm [3] since most of the findings in our study indicated moderate and massive splenomegaly. Additionally, the prevalence of splenomegaly was comparable to that in a study performed in Pakistan [17], in which the prevalence was 89.3%. Furthermore, these findings are consistent with those of a study from India [19], which reported incidences of 62%, 22.2% and 15.6% for massive, moderate and mild, respectively. The difference in moderate and mild symptoms may be due to the duration of symptoms among the study participants, and the sample size could be the reason since our sample size was larger than that of their study participants.

Of the 251 confirmed CML patients, approximately 86.1% were confirmed by FISH and were Ph+ (t (9; 22) positive). Approximately 9.2% and 4.8% of the patients were confirmed by the Xpert BCR-ABL Ultra test and PCR, respectively, which indicated that these patients were BCR::ABL1  or p210-positive. These molecular testing methods were also used in other studies; for example, *Ngono AP et al.*, from Cameroon, used (22.7%) FISH and (4.5%) RT‒PCR and detected the presence of the t(9; 22) or BCR::ABL1 transcript in all patients [1]. According to the CML data obtained by both quantitative and qualitative PCR, all the detected transcripts were of major breakpoint proteins, and p210 is encoded by the e13a2/e14a2 isoform. The presence of the BCR::ABL1 transcript as a result of reciprocal translocation between chromosomes 9 and 22 is well known in CML. In more than 95% of CML patients, the typical BCR::ABL1 transcript subtypes are e13a2 (b2a2), e14a2 (b3a2), or the simultaneous expression of both) [24, 25].

In our case, qualitative PCR was performed for FISH negative patients; accordingly, six patients who were FISH negative were P210-positive according to qualitative PCR. Qualitative PCR is appropriate for the initial diagnosis of CML, and it can detect the presence of the p210, p190 and p230 transcripts. Thus, although there is an unaffordability of different advanced molecular techniques among suspected CML patients, using these different molecular techniques is important for providing accurate diagnosis and early detection and for monitoring CML patients.

### Strengths and limitations

This study included relatively large sample sizes and was conducted at the only CML clinic in the country; thus, it could represent the country of interest, Ethiopia. Since this was the first study conducted on this topic, it provides current information on the hematological, clinical, cytogenetic and molecular profiles of newly diagnosed CML patients. As a limitation, further testing was not performed, as was clinical chemistry. In addition, for every patient, cytogenetic and molecular analyses were not performed due to the unavailability and unaffordability of these tests since they were performed abroad.

## Conclusion

During presentation, most of the CML patients had hyperleukocytosis, neutrophilia or anemia at TASH, Addis Ababa, Ethiopia. Patients in younger age groups with a median age of 36 years and more than 91% ≤ 60 years were affected by CML in Ethiopia. Approximately 85% of the CML patients had CP-CML, whereas more than 42.6% of the patients had abnormal platelet counts. Fatigue, abdominal pain, splenomegaly and weight loss were the most common signs and symptoms observed in the CML patients. Most of the study participants were Ph + according to FISH analysis, and the major breakpoint protein p210, which is encoded by the e13a2/e14a2 transcript, was detected in all patients diagnosed by both quantitative and qualitative PCR.

Most of the patients come from distant areas; as a result, they arrive after advanced stages with different signs and symptoms, thus better to decentralize the service to peripheral health facilities. In addition, a locally established reference range for hematological and clinical parameters should be established considering age, sex, geographical location and other variables, as well as access to advanced laboratory facilities for early detection and monitoring of cytogenetic and molecular parameters of CML patients.

## Data Availability

The datasets generated and/or analyzed during the current study are not publicly available due [we do not have consent from all patients and ethical approval committees to publish this data] but are available from the corresponding author on reasonable request.
